# Molecular dynamics simulations of Piezo1 channel opening by increases in membrane tension

**DOI:** 10.1016/j.bpj.2021.02.006

**Published:** 2021-02-12

**Authors:** Dario De Vecchis, David J. Beech, Antreas C. Kalli

**Affiliations:** 1 Leeds Institute of Cardiovascular and Metabolic Medicine, School of Medicine, University of Leeds, Leeds, United Kingdom; 2 Astbury Centre for Structural Molecular Biology, University of Leeds, Leeds, United Kingdom

## Abstract

Piezo1 is a mechanosensitive channel involved in many cellular functions and responsible for sensing shear stress and pressure forces in cells. Piezo1 has a unique trilobed topology with a curved membrane region in the closed state. It has been suggested that upon activation Piezo1 adopts a flattened conformation, but the molecular and structural changes underpinning the Piezo1 gating and opening mechanisms and how the channel senses forces in the membrane remain elusive. Here, we used molecular dynamics simulations to reveal the structural rearrangements that occur when Piezo1 moves from a closed to an open state in response to increased mechanical tension applied to a model membrane. We find that membrane stretching causes Piezo1 to flatten and expand its blade region, resulting in tilting and lateral movement of the pore-lining transmembrane helices 37 and 38. This is associated with the opening of the channel and movement of lipids out of the pore region. Our results reveal that because of the rather loose packing of Piezo1 pore region, movement of the lipids outside the pore region is critical for the opening of the pore. Our simulations also suggest synchronous flattening of the Piezo1 blades during Piezo1 activation. The flattened structure lifts the C-terminal extracellular domain up, exposing it more to the extracellular space. Our studies support the idea that it is the blade region of Piezo1 that senses tension in the membrane because pore opening failed in the absence of the blades. Additionally, our simulations reveal that upon opening, water molecules occupy lateral fenestrations in the cytosolic region of Piezo1, which might be likely paths for ion permeation. Our results provide a model for how mechanical force opens the Piezo1 channel and thus how it might couple mechanical force to biological response.

## Significance

Cells have sophisticated molecular machines that help them to respond to external mechanical forces. One of these machines is the membrane protein Piezo1. Piezo1 has a critical role in the circulatory system and tissue development and is associated with human diseases and disorders such as hemolytic anemia. For this reason, it is critical to understand how Piezo1 functions at the molecular level. In this study, we use computer simulations to study Piezo1 activation. Our studies suggest that Piezo1 adapts to the stretch of the membrane by flattening its structure and reducing its membrane indentation. Hence, we propose a model that explains the steps by which Piezo1 channels may adapt and open in response to increased membrane tension.

## Introduction

Piezo1 is a critical mechanical sensor that is found in endothelial cells, red blood cells, and other cell types (1, 2, 3). It plays a critical role in the circulatory system and tissue development. Mutations in Piezo1 are linked to human diseases such as lymphedema (2,4) and hematological disorders such as hemolytic anemia (5) and resistance to malaria (6). Its main function is to permeate ions, including Na^+^ and Ca^2+^ in response to mechanical stimuli (3,7,8).

Structural data show that Piezo1 is a trimer (9, 10, 11, 12) with a trilobed topology and a curved membrane region that forms the blades (9, 10, 11, 12). On the intracellular side, each Piezo1 subunit has a beam region (i.e., an extended *α*-helix) that connects the periphery of Piezo1 with its central region. The three Piezo1 subunits converge to a central pore region with a C-terminal extracellular domain (CED) between each of the last two transmembrane (TM) helices. The pore region of Piezo1 is surrounded by three blades. Each blade consists of nine four-*α*-helix bundles; the three N-terminal bundles are not present in this structural data for Piezo1.

It has been shown that because of its unique curved membrane region (i.e., the blade region), Piezo1 induces a dome in the membrane (11) when Piezo1 is in its closed state. Mechanical calculations suggested that in addition to the local dome, Piezo1 creates a wider footprint in the membrane that extends beyond the Piezo1 radius (13). This extended footprint may regulate Piezo1 sensitivity to mechanical force (13). Upon activation, Piezo1 was shown to adopt a flatter conformation (14,15); however, the molecular details of Piezo1’s active conformation as well as its intermediate structural states during activation are unknown. Flattening of Piezo1 during activation may be the result of the in-plane area expansion (11), as also seen for MscL mechanosensitive channel (16). Hypotheses for Piezo1 gating include the “force-from-lipids” principle (17,18), which suggests that Piezo1 senses mechanical forces through the bilayer (1,19) and a direct involvement of the cytoskeleton as well as the extracellular matrix in Piezo1 activation (20). However, the molecular and structural changes underpinning the Piezo1 gating mechanism and how the channel senses forces in the membrane remain unknown.

In silico methodologies have been successfully used to study membrane mechanics under stress (21,22), including for Piezo1 (15). However, although *μ*s-long atomistic simulation of a partial Piezo1 structure at a physiological negative pressure detected local fenestrations, such simulation did not record a full opening event (15). Indeed, the activation time of mechanosensitive channels is in the order of milliseconds (23,24), which is currently challenging with atomistic molecular dynamics (MD) simulations.

In this study, we used MD simulations and comparison with available knowledge to suggest the structural rearrangements that occur when Piezo1 progresses from a closed to an open state when increasing mechanical tensions are applied to a membrane bilayer. Our results suggest a relationship between membrane mechanics and Piezo1 structural rearrangements and show that upon stretching Piezo1 moves to a flatter stable conformation propelled by the simultaneous expansion of its blade regions. We also show the critical role that the blade regions play in sensing tension forces in the membrane. Piezo1 flattening and expansion result in an increase of the in-plane area and changes in the beam helix tilt angle. Our Piezo1 flat open model also shows solvated lateral fenestrations in the cytosolic region of Piezo1, which might be likely paths for ion permeation. These events lead in turn to the opening of the channel and to the migration of lipids outside of the initially occupied Piezo1 central pore region, suggesting structural changes that happen during Piezo1 mechanical activation.

## Materials and methods

### Modeling the Piezo1 trimer

Structural data were obtained from the cryogenic electron microscopy (cryo-EM) structure Protein Data Bank, PDB: 6B3R (11). Missing residues were added with MODELER (v 9.19) (25), and the loop refinement tool (26) was used to remove a knot in one chain between residues 2066 and 2074. The best loop was selected out of 10 candidates according to the discrete optimized protein energy method (27). The final Piezo1 model does not include the first 576 residues because they are not present in the template and residues 718–781, 1366–1492, 1579–1654, and 1808–1951, which are exposed to the cytosol. Therefore, each chain is composed by five nonoverlapping fragments: residues 577–717, 782–1365, 1493–1578, 1655–1807, and 1952–2547. This Piezo1 model was also used to prepare the two blade-free constructs comprising of residues 1973–2547 (Piezo1^Δ1973^) and 2104–2547 (Piezo1^Δ2104^). Piezo1^Δ1973^ has only the last four-helical bundle which is the closest bundle to the channel pore. Piezo1^Δ2104^ is a complete blade-free Piezo1.

### Coarse-grained simulations

The sizes and compositions of all the simulated systems are listed in Table S1. For all the Piezo1 models described above, the protein coordinates were converted to a coarse-grained (CG) resolution and energy minimized in a vacuum with GROMACS 2016 (Fig. S1) (28). Fig. S1 shows that despite the minimization in a vacuum, no conformational changes were observed within Piezo1 during the minimization step. The CG-MD simulation was essential to equilibrate the lipid bilayer around the protein and reconstitute the Piezo1 membrane indentation. The CG-MD simulations were performed using the Martini 2.2 force field (29) and GROMACS (28). To model the protein secondary and tertiary structure, an elastic network model with a cutoff distance of 7 Å was used. For the equilibration simulation Piezo1 model was inserted in a complex asymmetric bilayer using the INSert membrANE tool (30). Three independent system assembly steps and as many CG equilibrations were carried out, the first of which were used in this study (see Table S1, Equil 1). During the CG-MD simulations, the protein was position restrained, and thus, no changes within the protein were allowed. With the exception of the simulation carried out in a 1,2-dilauroyl-sn-glycero-3-phosphocholine (DLPC) (Piezo1^DLPC^) bilayer, the composition of the model bilayer was the following: for the outer leaflet, 1-palmitoyl-2-oleyl-phosphtidylcholine (POPC) 55%, sphingomyelin (SM) 5%, 1-palmitoyl-2-oleyl-phosphtidylethanolamine (POPE) 20%, and cholesterol 20%. For the inner leaflet, POPC 50%, POPE 20%, 1-palmitoyl-2-oleyl-phosphtidylserine (POPS) 5%, cholesterol 20%, and phosphatidylinositol 4,5-bisphosphate (PIP_2_) 5%. The system was neutralized with a 150 mM concentration of NaCl. The model was further energy minimized and subsequently equilibrated for 500 ns with the protein particles restrained (1000 kJ · mol^−1^ · nm^−2^) to allow the membrane bilayer to equilibrate around the model. The time step for all the CG simulations was 20 fs. The equilibration was performed at 323 K, with protein, lipids, and solvent separately coupled to an external bath using the v-rescale thermostat (31) (coupling constant of 1.0). The temperature of 323 K is above the transition temperatures of all lipid species in the systems, therefore avoiding the lipids to undergo phase transitions to the gel phase. Pressure was maintained at 1 bar (coupling constant of 1.0) with semi-isotropic conditions and compressibility of 3 × 10^−6^ using the Berendsen barostat (32). Lennard-Jones and Coulombic interactions were shifted to 0 between 9 and 12 Å and between 0 and 12 Å, respectively.

### Atomistic simulations

After the CG-MD equilibration (Equil 1 in Table S1), two POPC molecules were removed because the headgroup was trapped between Piezo1 TM bundles. Moreover, to restore membrane asymmetry, three molecules of PIP_2_ were removed because they flipped to the outer leaflet. The number of lipids removed is extremely small compared with the number of lipids in each leaflet (∼1300 phospholipids and ∼300 cholesterol molecules in each leaflet), and thus, it did not introduce any bias during our simulations. The system obtained was minimized and further converted to atomistic resolution as described in (33). A moderate concentration of 3.0 mM of Ca^2+^ (i.e., 50 ions) was added to the box, and neutrality was restored with counterions. The Ca^2+^ was added because Piezo1 was also shown to be permeable to Ca^2+^. The obtained system was energy minimized and subsequently equilibrated in four NPT ensemble runs of 20,000 steps each with an increasing time step from 0.2 to 2 fs, and the protein particles were restrained (1000 kJ · mol^−1^ · nm^−2^). A further equilibration step of 5 ns with a time step of 1 fs and C*α* atoms restrained (1000 kJ · mol^−1^ · nm^−2^) was performed to relax the Piezo1 model embedded in a highly curved bilayer (i.e., the membrane indentation). Very quickly in the equilibration, quantities such as box size and volume reached a value and remained in that value for the rest of the equilibration. Stretch-induced conformational changes in the Piezo1 model were investigated by NPT ensemble unrestrained simulations of 50 ns in which the bilayer plane (*x*-*y* plane) pressure was varied semi-isotropically between −40, −30, −20, −5, and +1 bar, whereas the pressure in bilayer normal (*z*) direction was kept at +1 bar. The +1- and −30-bar systems were further extended to 100 ns. A similar approach was used to investigate structural transitions for the TREK-2 mechanosensitive channel (34). All the atomistic systems were simulated using GROMACS 2016 (28) with CHARMM36 force field (35)⁠ and a 2-fs time step. A Berendsen semi-isotropic pressure coupling (32) at 1 bar was used during all the equilibration phases. The Parrinello-Rahman barostat (36) was further used for the stretch-induced simulations. All simulations were performed at 323 K, with protein, lipids, and solvent coupled to an external bath using the v-rescale thermostat. Such temperature was also used in simulations of other mechanosensitive channels (34). Long-range electrostatics were managed using the particle-mesh Ewald method (37). The LINCS algorithm was used to constrain bond lengths (38).

The same protocols described above were followed for all the all-atom simulations (see Table S1), with the exception of the Piezo1^−40.rep1^ and Piezo1^−40.rep2^ systems. For these systems, the protein coordinates from the last frame from each of the two repeat simulations at −40 bar were inserted in a model asymmetric membrane by using the CHARMM-GUI webserver (39). The protein was embedded in a bilayer with the same composition as described above. To reconstitute the Piezo1 open pore, lipids present in the channel mouth were removed to allow the water to flow and fill the Piezo1 open pore. For the Piezo1^−40.rep1^, we removed four molecules of cholesterol, seven of POPC, and three of POPE over a total of 956, 2516, and 957 lipids, respectively. For the Piezo1^−40.rep2^, we removed five molecules of cholesterol, eight of POPC, and two of POPE over a total of 951, 2504, and 954 lipids, respectively. The same concentrations of Ca^2+^ were added to each system as above (3.0 mM), subsequently neutralized with counterions, and further minimized. For each system, two NPT equilibrations were performed of 2 and 8 ns with 1 and 2 fs, respectively. The systems consisted of ∼3 million atoms (see Table S1). Protein backbone atoms were restrained in both equilibrations (1000 kJ · mol^−1^ · nm^−2^), whereas only in the first equilibration, the phosphorous atoms from the phospholipids and the oxygen atoms from the cholesterol were restrained (1000 kJ · mol^−1^ · nm^−2^) to allow the water to fill the Piezo1 open pore once the lipids were removed from the channel mouth (see above). Piezo1^−40.rep1^ and Piezo1^−40.rep2^ systems (see Table S1) were run at +1 bar for 100 ns as described above for the other systems.

### Analysis

To calculate the area of the box, the tool gmx energy from GROMACS 2016 (28) was used to extract the *x*-side and the *y*-side of the simulated box for each frame. The area was subsequently calculated by multiplying these values as *x*-side × *y*-side. The root mean-square deviation (RMSD) was calculated on the protein C*α* using the gmx rms tool from GROMACS 2016 (28). For each system the initial coordinates were considered as a reference structure. All the errors shown are the standard deviation. The in-plane projection of the Piezo1 membrane indentation (A_proj_) was calculated as follows: the program visual molecular dynamics 1.9.3 (VMD) (40) (http://www.ks.uiuc.edu/Research/vmd/) was used to calculate distances between the C*α* atom from residue Ala641, located within the first bundle embedded in the bilayer from each Piezo1 chain and for each trajectory. The distance was then used to calculate the radius *r* of the circumscribed circle and considering the Piezo1 triskelion as the vertices of a scalene triangle that is planar to the surface of the membrane bilayer using an in-house script according to Eq. 1.

(1) $$r=\frac{\left(a\times b\times c\right)}{\sqrt{\left(a+b+c\right)\left(b+c-a\right)\left(c+a-b\right)\left(a+b-c\right)}}.$$

The values *a*, *b*, and *c* are the sides of the scalene triangle. The area of the circumference was then calculated as *π* × *r*^2^. For the radius *r* and the A_proj_, the standard deviation was calculated. The average partial density was calculated using the GROMACS 2016 (28) tool gmx density. The two-dimensional density maps for lipids and cholesterol were calculated using gmx densmap. The tilting angle relative to the bilayer normal and distance from the Piezo1 pore of the Piezo1 beams, pore-lining helices 38 and 37, were calculated using gmx bundle and gmx distance tools from GROMACS 2016 (28). For all calculations, the Piezo1 pore region comprised the same residues that were used for the calculation of the cavity (see below). The tool trj_cavity (41) (https://sourceforge.net/projects/trjcavity/) was used for the calculation of the volume of the Piezo1 pore cavity as well as for the water molecules that overlap to it. The considered residues were 2450–2547, 2214–2224, and 2326–2334. The trajectories were fitted on the C*α* of the considered residues before the calculation and options dim 4 (i.e., the degree of how buried they were) and spacing 1.4 (i.e., the size of the grid voxel in Å) were used. Salt bridges were calculated with VMD 1.9.3 (40) using a 6-Å cutoff and, when indicated, without considering the first 30 ns of simulation. For the calculation of the area per lipid (APL), the tool FATSLiM (42) (http://fatslim.github.io/) was used. The lateral pressure *P*_*L*_ was calculated as explained for the TREK-2 mechanosensitive channel (34). Molecular graphics were done using VMD 1.9.3 (40). Data were plotted using Grace (https://plasma-gate.weizmann.ac.il/Grace/).

## Results and discussion

To investigate how mechanical forces act on the Piezo1 channel, we simulated the recent Piezo1 structure solved by cryo-EM in a model membrane using a serial multiscale MD simulation approach. Missing residues were added to the structure before the simulations (see Materials and methods). Initial CG-MD simulations of 500 ns was carried out to equilibrate the bilayer around Piezo1 and observe the unique Piezo1 footprint suggested previously (11,14,43). The asymmetric bilayer used in our simulations mimics the native endothelial membrane (44) (see Materials and methods), which is one of the systems that Piezo1 functions in physiologically (3,45). Three independent CG system assemblies followed by three independent equilibration runs were carried out (see Materials and methods), and in all cases, the timescale of the CG simulations (500 ns) was enough for the dome to be formed around Piezo1 curved blades with a depth of ∼6 nm in agreement with other studies (11). Although the CG approach was critical for creating the Piezo1/membrane system, the elastic network implemented in the CG approach does not allow us to fully study the Piezo1 pressure-dependent dynamical responses. For this reason, the system was backmapped to an atomistic resolution and further simulated in independent repeat simulations applying pressure to the bilayer (i.e., along *x* and *y* axes) ranging from the native-like pressure of +1 to −40 bar, as described in Materials and methods. Under these conditions, we were able to calculate the lateral pressure (*P*_*L*_) applied on the bilayer, which is 14.2 mN/m for the −5 bar, 45.8 mN/m for the −20 bar, 59 mN/m for the −30 bar, and 67.8 ± 0.35 mN/m for the −40 bar system. The tension required for the half-maximal activation of Piezo1 is 2.7 ± 0.1 or 4.7 ± 0.3 mN/m for outside-in and inside-out patches (46). The tensions in our simulations are higher than the experimentally derived half-maximal values and above the membrane rupture limit, but they were used to enable shorter timescales accessible for atomistic simulations.

### Tension causes Piezo1 to flatten and expand

The different pressures cause expansion of the simulated box to a different degree with the membrane in the system adapting to each pressure. This results in two main membrane perturbations: an increase of the APL in both leaflets for all systems (Fig. S2 *A*) and a thinning of the membrane bilayer (Fig. S2 *B*); the APL and the membrane thickness for the system with the highest tension in the bilayer (i.e., −40 bar) were 1 nm^2^ and ∼2.7 nm, respectively (Fig. S2). A similar approach revealed that an increase of the APL resulted in the transition of the TREK-2 mechanosensitive channel from a down to an up conformation (34). We note that in all tensions, within the simulated time, the bilayer is still in an intact state (i.e., no water moves through the bilayer).

Our analysis above shows that the APL reaches a plateau for both leaflets within the first 10 ns of the simulation (Fig. S2). Two of our systems, the −30 bar and the +1 bar, were extended up to 100 ns. In both cases, the analysis confirms that no further change in the APL occurs with increased time (Fig. S2).

**Figure 1:**
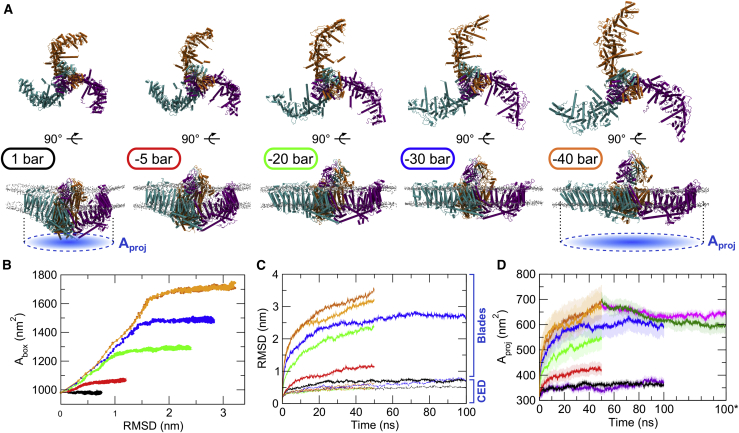
Piezo1 channel flattens, and its CED becomes exposed in response to increased membrane tension. For each system, the applied negative pressure in each simulation is indicated with different colors: black, 1 bar; red, −5 bar; green, −20 bar; blue, −30 bar; and orange, −40 bar. (*A*) Shown are snapshots of the last frame from simulations of Piezo1 embedded in a model membrane bilayer. (*B*) Given is the correlation between the area, i.e., *x*-side × *y*-side, of the simulated box (A_box_) and the RMSD calculated using the protein C*α* of the Piezo1 blades. (*C*) Shown is the RMSD calculated using the protein C*α* of the CED, dashed lines (lower values), and of the rest of the protein (i.e., the Piezo1 blades) and straight lines (higher values). (*D*) Shown is the A_proj_ (indicated by a *dashed blue circle* in *A* as a function of time for all the systems; see Materials and methods). The timeline for the Piezo1^DLPC^, Piezo1^−40.rep1^, and Piezo1^−40.rep2^ systems (values translated by 50 ns to 100∗ ns for clarity) is shown in purple, magenta, and dark green, respectively. For the two repeat simulations at −40 bar, the timelines are colored in orange (first repeat) and dark orange (second repeat). The transparent regions show the standard deviation. To see this figure in color, go online.

The Piezo1 structure showed a remarkable adaptation to a stretched bilayer, with its N-terminal blades remaining embedded within the bilayer during all simulations and extending toward flatter conformations (Fig. 1 *A*). This result is in good agreement with findings by Lin et al., who used atomic force microscopy to show that application of mechanical force results in flatter conformations of Piezo1 (14). The degree of flattening was different for the different tensions, with the system having the highest tension resulting in an almost flat Piezo1 structure (Fig. 1 *A*). This suggests that Piezo1 flattening follows the bilayer flattening and not the contrary because within the timescales of our simulations, the channel seems to adapt its curvature to accommodate the changes in the bilayer that result from different tensions. Therefore, we suggest that it is not the Piezo1 opening leading to flattening of the membrane, but it seems that Piezo1 blades sense the force that comes from the bilayer.

**Figure 2:**
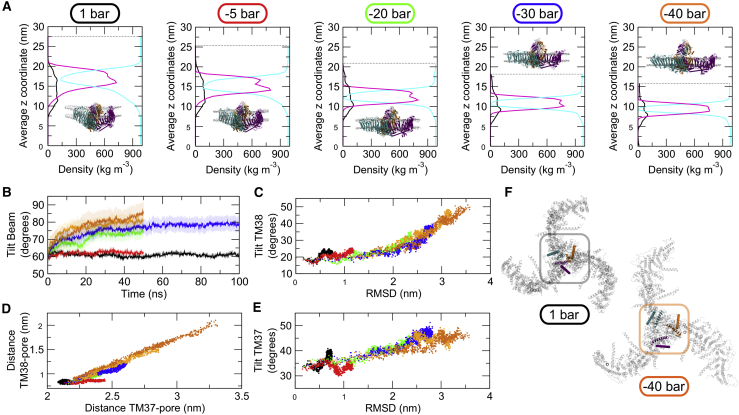
Coordinated tilting of Piezo1 beams and ion pore helices in response to increased membrane tension. (*A*) Given is the partial density profile along the *z* direction (that is perpendicular to the bilayer *x*, *y* plane) for the protein (*black line*), the membrane (*magenta line*), and the solvent (i.e., water and ions; *cyan line*). Values from the first repeat at −40 bar are shown. Insets are the last frames from simulations, duplicated from Fig. 1*A* and for illustrative purpose only. (*B*) Shown is a tilt angle relative to the bilayer normal for the beam helix. The transparent regions show the standard deviation. (*C*) Shown is the correlation between the RMSD of the Piezo1 blades and the tilt angle relative to the bilayer normal of the TM38 helix. (*D*) Shown is the correlation between the distances from the center of mass of the TM38 or TM37 helices and the Piezo1 pore. (*E*) Shown is the correlation between the RMSD of the Piezo1 blades and the tilt angle relative to the bilayer normal of the TM37 helix. (*F*) Given are snapshots of the last frame from the +1- and −40-bar systems with the Piezo1 shown from the extracellular side. The center is highlighted with a box. The TM38 (*cartoon*) and the TM37 (*ribbon*) from each Piezo1 chain are also shown. Membrane and solvent are not displayed. The color code for the applied negative pressure in each simulation is the same as in Fig. 1. To see this figure in color, go online.

Fig. 1 *B* shows the correlation between the area of the simulated box and the RMSD calculated using the C*α* atoms of the blades, demonstrating that they both increase until the bilayer stops expanding. RMSD analysis showed that although the blade bundles remain intact during stretching, they are the major contributors to the RMSD drift, whereas the RMSD calculated over the C*α* atoms of the CED is comparable between the different applied negative pressures (Fig. 1 *C*). In the bilayer without tension (+1 bar), Piezo1 maintained its curved shape even though the simulation was twice as long (i.e., 100 ns) compared with some of the other simulations with stretch applied to the bilayer. This shows that the curved shape of Piezo1 blades forces the bilayer to adopt a curved trilobed shape. It also confirms that our CG simulations were sufficient to create a stable dome around Piezo1 when no tension was applied to the bilayer. Interestingly, although Piezo1 reaches a flat conformation in both the −30- and −40-bar systems, larger conformational changes with more opening of the pore were observed in the −40-bar simulations. Even when the −30-bar systems were extended up to 100 ns (compared with 50 ns of the −40 bar), the structural changes in this system were smaller (Fig. 1, *A* and *B*). It should also be noted that although small, the −40- and −30-bar simulations have a somewhat different effect on the membrane topology. For example, the thinning observed in the −40-bar simulations are somewhat larger compared with −30 bar, and the APL is somewhat smaller in the −30-bar simulation. These changes, although small, result in different changes within the protein. Indeed, our simulations of Piezo1 at −30 and −40 bar do not have exactly the same effect on the protein. Piezo1 simulated at −30 bar does not reach a complete flat conformation as observed for the −40-bar simulations. The RMSD of the blades (Fig. 1 *C*) and the projected area (Fig. 1 *D*) do not have the same values. Additionally, in the −40-bar simulations, the beam helices reached almost 90° (Fig. 2 *B*), and the CED becomes more exposed (Fig. 2 *A*).

As indicated above, the structure we simulated is missing its three N-terminal four bundles because these parts were not resolved in cryo-EM studies, possibility because of their high flexibility. Because of the importance of the blade structures in the mechanosensitivity of Piezo1, this partial Piezo1 structure (i.e., with fewer N-terminal blade bundles) might not have the same absolute force sensitivity as the full-length Piezo1 structure. This could explain why higher membrane tension values were required compared with those suggested from studies of Piezo1 overexpressed in HEK293t cells (46).

**Figure 3:**
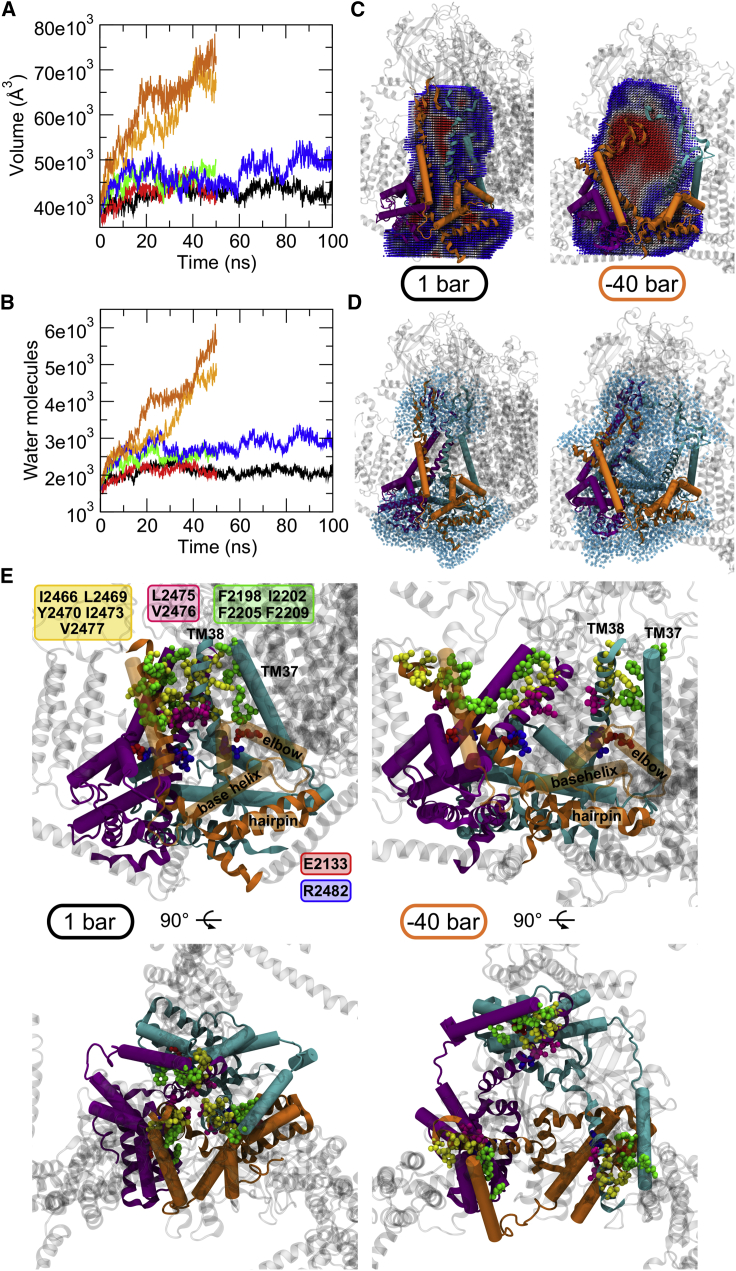
Expansion of the Piezo1 pore region in response to increased membrane tension. (*A*) Shown is the volume of the cavity of the Piezo1 pore region as a function of time for each simulated system. The applied negative pressure in each simulation is indicated with different colors: black, 1 bar; red, −5 bar; green, −20 bar; blue, −30 bar; and orange, −40 bar. (*B*) Shown are the number of water molecules overlapping with the calculated cavity in (*A*) as a function of time for each simulated system. (*C*) Given is the volume of the cavity within the core of the Piezo1 structure for the +1- and −40-bar systems. The structures are the last frames from each simulation and focus only on the Piezo1 pore region. For the −40-bar system, the second repeat simulation is shown. The cavity (sliced) is shown as spheres and colored according to the occupancy during the trajectory, from less populated (*blue*) to more populated (*red*). Residues selected for the calculation of the cavity are shown in ribbon (including the TM38 helix; see Materials and methods). TM37, elbow and base helix are represented as cartoon cylinders. The Piezo1 chains are shown in the same colors as in Fig. 1*A*. (*D*) Water molecules (*light cyan transparent spheres*) within 8 Å from the residues selected for the calculation of the cavity for the +1- and −40-bar systems. The structures are the same shown in (*C*). (*E*) Detail of the Piezo1 channel mouth for the +1 bar and the −40 bar systems shown from side-on (*above*) and extracellular helicopter (*below*) views. The structures are the same as presented in (*C*) and (*D*) and show the hydrophobic interactions between the pore-lining helices TM38 (*yellow residues*) and the TM37 (*green residues*). The salt bridge between the elbow and the TM38 from a neighboring subunit is also shown (*red* and *blue residues*). Residues from the Piezo1 hydrophobic gate (residues L2475 and V2476) are in magenta. To see this figure in color, go online.

We investigated further if the large conformational change we observed for the blades under large tension are needed for Piezo1 to sense mechanical force. To this end, we simulated two Piezo1 blade-free constructs: the Piezo1^Δ1973–2547^, which consists of the four-helical bundle that is the most proximal to the pore, and the Piezo1^Δ2104–2547^, which does not have any of the nine four-helical bundles (see Materials and methods). Both constructs were simulated at −40-bar pressure for 100 ns each to investigate if the same tension that we saw to open Piezo1 would also open a Piezo1 blade-free channel. Piezo1^Δ1973–2547^ and Piezo1^Δ2104–2547^ have a similar behavior and did not open at −40-bar pressure, as indicated by the RMSD of the inner pore (Fig. S3 *A*) and the volume of the cavity of the Piezo1 pore region (Fig. S3 *B*). The value of the volume of the cavity in the Piezo1 blade-free constructs is similar to the values observed when Piezo1 was simulated at 1 bar (Fig. 3 *A*). We also note that for both constructs (Piezo1^Δ1973^ and Piezo1^Δ2104^), the APL and the bilayer thickness are comparable with our previous simulations at −40 bar (Fig. S2, *A* and *B*), but these conditions are not sufficient to open the pore of a Piezo1 blade-free system. Moreover, a feature of our blade-free Piezo1 models resides in their inability to create the Piezo dome (the membrane indentation), which was suggested to be necessary for the Piezo1 mechanosensitivity (11). Overall, our data about the Piezo1 blade-free constructs show that the blade domain is the region of Piezo1 that senses mechanical stress (10). Without this region, Piezo1 does not seem to be able to sense mechanical force.

Upon flattening of its blades, Piezo1 expands, thus increasing the A_proj_ (Fig. 1 *A*). During this process, no unfolding events within the protein were evident. This is in good agreement with recent studies that also suggested an increase of the A_proj_ involved in the Piezo1, and recently Piezo2, activation (11,14,43). The A_proj_ can be approximated by a projected circle on the membrane plane (Fig. 1 *A*). By first approximating the Piezo1 triskelion to the vertices of a scalene triangle, we determined the circumscribed circle (i.e., the A_proj_) as described in Eq. 1 (see Materials and methods). Our stretch simulations are indicative of a concurrent movement of the Piezo1 blades, which all flatten synchronously (Videos S1 and S2). Fig. 1 *D* shows how the increase of applied mechanical force during simulations (e.g., more tension) causes an increase of the A_proj_ value, which reaches ∼680 nm^2^ at −40 bar (Fig. 1 *D*, *orange data*). Recent cryo-EM structural data of the full-length Piezo2 estimated a A_proj_ of the dome to 700 nm^2^ (43). Although the current value for the full-length Piezo1 is still unknown, this agreement suggests that upon flattening, our incomplete Piezo1 model covers the area that is expected to be within the dome in the closed state calculated for the full-length Piezo2 (43). This is in good agreement with the suggestion that the dome provides an energy store for Piezo1 activation (11). A small increase of A_proj_ was observed in the +1-bar simulation (Fig. 1 *D*), suggesting that Piezo1 adapts slightly when inserted in a membrane environment in respect to the cryo-EM structure.

Video S1. Stretch MD simulation causes flattening of the Piezo1 membrane indentation The first repeat of the −40-bar simulation is shown here. The Piezo1 chains are indicated in ribbon representation and colored in orange, purple, and cyan. Phosphate atoms from the phospholipids are depicted as blue spheres. Solvent molecules have been removed for clarity.

Video S2. Stretch MD simulation causes the in-plane expansion of Piezo1 The top view of the first repeat of the −40-bar simulation is shown here. The Piezo1 chains are indicated in ribbon representation and colored in orange, purple, and cyan. Phosphate atoms from the phospholipids are depicted as blue spheres. Solvent molecules have been removed for clarity.

As discussed above, upon tension application, we observed thinning of the bilayer. To investigate whether bilayer thinning can result in Piezo1 activation, we ran a 100-ns simulation with Piezo1 embedded in a DLPC bilayer at 1-bar pressure (Piezo1^DLPC^; see Table S1 and Materials and methods). In this simulation, the bilayer thickness is similar to the values obtained in our −20-bar stretch simulation (Fig. S2 *B*). However, the channel remains closed, its blades are curved, and the membrane dome remains as it is for the 1-bar system (Fig. S4 *C*). The RMSD calculated over the C*α* trace for the inner pore and for the full protein both confirm that the channel remains closed (Fig. S4 *A*). In agreement with that, the volume of the cavity of the Piezo1 inner pore has similar values with the +1-bar simulation (Fig. S3 *B*). This suggests that although thinning is observed during tension application, it is not sufficient to change Piezo1 shape.

In addition to a structural model of a flat Piezo1, our stretched simulations allowed us to start investigating how the Piezo1 flattened state would adapt once the mechanical tension stops. This likely represents a very early postactivation state. To do that, we extracted the protein coordinates from the last frame from our −40-bar systems and carried out further two all-atom MD simulations of 100 ns each at 1-bar pressure (Piezo1^−40.rep1^ and Piezo1^−40.rep2^; see Materials and methods). Calculation of the RMSD of the pore and of the whole protein (Fig. S5, *A* and *B*) shows that for both repeats Piezo1^−40.rep1^ and Piezo1^−40.rep2^, the channel mouth is still open, and the channel is flat (Fig. S5 *C*), therefore confirming the stability of our Piezo1 flat structure. In addition, calculation of the A_proj_ (Fig. 1 *D*) showed that although this value slightly decreased (because no membrane tension is applied), the A_proj_ moderately remains above the values obtained for the −30-bar system after 100 ns.

To reconstitute the solvated open pore, because it was observed for the −40 bar simulations, lipids that occupied the channel mouth were removed after the Piezo1^−40.rep1^ and Piezo1^−40.rep2^ system assembly (see Materials and methods; Fig. S5 *D*). Interestingly phospholipids progressively start to reoccupy the Piezo1 open channel mouth after the first 30 ns and finally block the Piezo1 pore (Fig. S5 *D*). As a result, water is no longer able to go through the Piezo1 pore. Even though our protocol might have some limitations, e.g., few lipids were removed from the channel mouth, it suggests that lipids re-enter the channel mouth as soon as the tension is removed from the simulations. This may represent a state of Piezo1 in the very early stage of the inactivation. This might also offer a possible molecular explanation on how lipids re-entering the channel mouth might contribute to the rapid decay of the Piezo-mediated current while the stimulus is still present (47).

Overall, our approach provides novel molecular insights, to our knowledge, into the activation dynamics of Piezo1 channel in a native-like lipid environment. Moreover, we dynamically quantify the different A_proj_ expansions under different mechanical stress and the area covered by a flat, activated Piezo1 channel. Our studies also reveal the critical role of Piezo1 blade region in sensing of mechanical force and suggest that membrane thinning is not sufficient to activate Piezo1 in the absence of tension. Finally, the extended structure obtained at −40 bar agrees with the evidence that positively correlate the size of the vesicle where Piezo1 is embedded with a flattened structure (14) and may also be needed for Yoda1 binding (15).

### Structural rearrangements in Piezo1 pore

Piezo1 flattening caused by membrane tension results in the displacement of the CED relative to the membrane plane (Fig. 1 *A*). CED is hidden within the dome at the beginning of the simulations or in the +1-bar system. Flattening of Piezo1 and of the bilayer results in the exposure of CED as it emerges outside the dome. The degree of CED exposure above the dome increases as the tension in the bilayer increases; in the −30- and −40-bar systems, CED is fully exposed. The density analysis revealed that a considerable portion of the Piezo1 structure remained consistently exposed to the cytoplasmic side even after the in-plane expansion (Fig. 2 *A*). It is possible that these cytoplasmic regions remain exposed to the cytosol during activation to provide a platform for Piezo1 interactions with the cytoskeleton, which is in agreement with the cytoskeleton’s suggested mechanoprotective role in Piezo1 function (19).

Another Piezo1 component that undergoes noticeable structural rearrangement is the beam helix (residues 1300–1365; Fig. 2 *B*). During the simulations with tension in the bilayer, the tilt angle of each Piezo1 beam changed from ∼65 to ∼85° until it is almost parallel to the bilayer when Piezo1 is in a flat conformation. The beam tilt movement is correlated with the change in the RMSD (Fig. S6 *A*), suggesting it may be coupled to Piezo1 flattening. The beam is a long *α*-helix exposed to the cytoplasm that underpasses the three C-terminal proximal bundles. Therefore, our data suggest that each Piezo1 beam may act as a set of levers and that the concerted movement of the beams during mechanical sensing contributes to gating.

We next sought to analyze the structural changes involving the pore-lining helices. Calculation of the tilt angle of the TM38 relative to the bilayer normal shows that higher negative pressure, and thus larger tension, corresponds to a higher tilting of the pore-lining TM38 (Fig. S6 *B*). The highest tilt angle is observed in the −40-bar system (Fig. 2 *C*). Calculation of the distance of TM38 relative to the pore center of mass also shows that larger tension corresponds to a larger displacement (Fig. S6 *C*). Therefore, during Piezo1 flattening and in-plane expansion, TM38 both increases its tilt angle and its distance from the pore center of mass. These two components are correlated with the changes in the RMSD calculated for the Piezo1 blades (Fig. 2 *C*; Fig. S6 *C*). This shows that TM38 displacement/tilting occurs while Piezo1 flattens and expands (Fig. 2 *F*). Similarly, the distance from the pore and tilting of the TM38 correlates with its antiparallel TM37 (Fig. 2, *D*–*F*; Fig. S6, *D* and *E*), suggesting a simultaneous lateral movement and tilting of both helices that might be the mechanism that results in the opening of the pore.

### Tension leads to Piezo1 channel opening

Calculation of the water-filled cavity in Piezo1 pore region shows that the volume of this cavity increases with the applied negative pressures in our simulations until it reaches ∼75,000 Å^3^ in the −40 bar (Fig. 3 *A*). The cavity is defined by the three TM38 and some regions of the CED (see Materials and methods) because the loops that connect this domain with the TM37/38 form a structure similar to a “cage” just above the channel mouth, which is characterized by a funnel shape (Fig. 3 *C*). Fig. 3 *A* shows that tension of −30 bar, although it contributes to flattening of the Piezo1 structure and almost maximizes the beam orientation, it is not sufficient to open the channel pore and maximize the volume of the cavity; the cavity in the −30 bar system is not hydrated as much as in the −40 bar (Fig. 3, *B* and *D*). Note that in the +1-bar system, the cavity was not increased, nor was the channel hydrated. After the pore hydration, our −40-bar simulations found that lateral fenestrations in the cytosolic region of Piezo1 are also hydrated (Fig. S7 *A*). Although we did not record complete ion passage events (likely because of the rather short simulation timescale), it is possible that these hydrated fenestrations may be part of the ion permeation pathway. Interestingly, the lateral fenestrations were present also at the beginning of the Piezo1^−40.rep1^ and Piezo1^−40.rep2^ simulations in which we stopped applying tension to the bilayer with the open Piezo1 structure, but they gradually disappear because lipids start to reoccupy the Piezo1 channel mouth (Fig. S5 *D*). We note, however, that during our simulations, sodium ions enter the extracellular side of the pore of the channel but without completely passing through (see Fig. S7, *B* and *C*).

**Figure 4:**
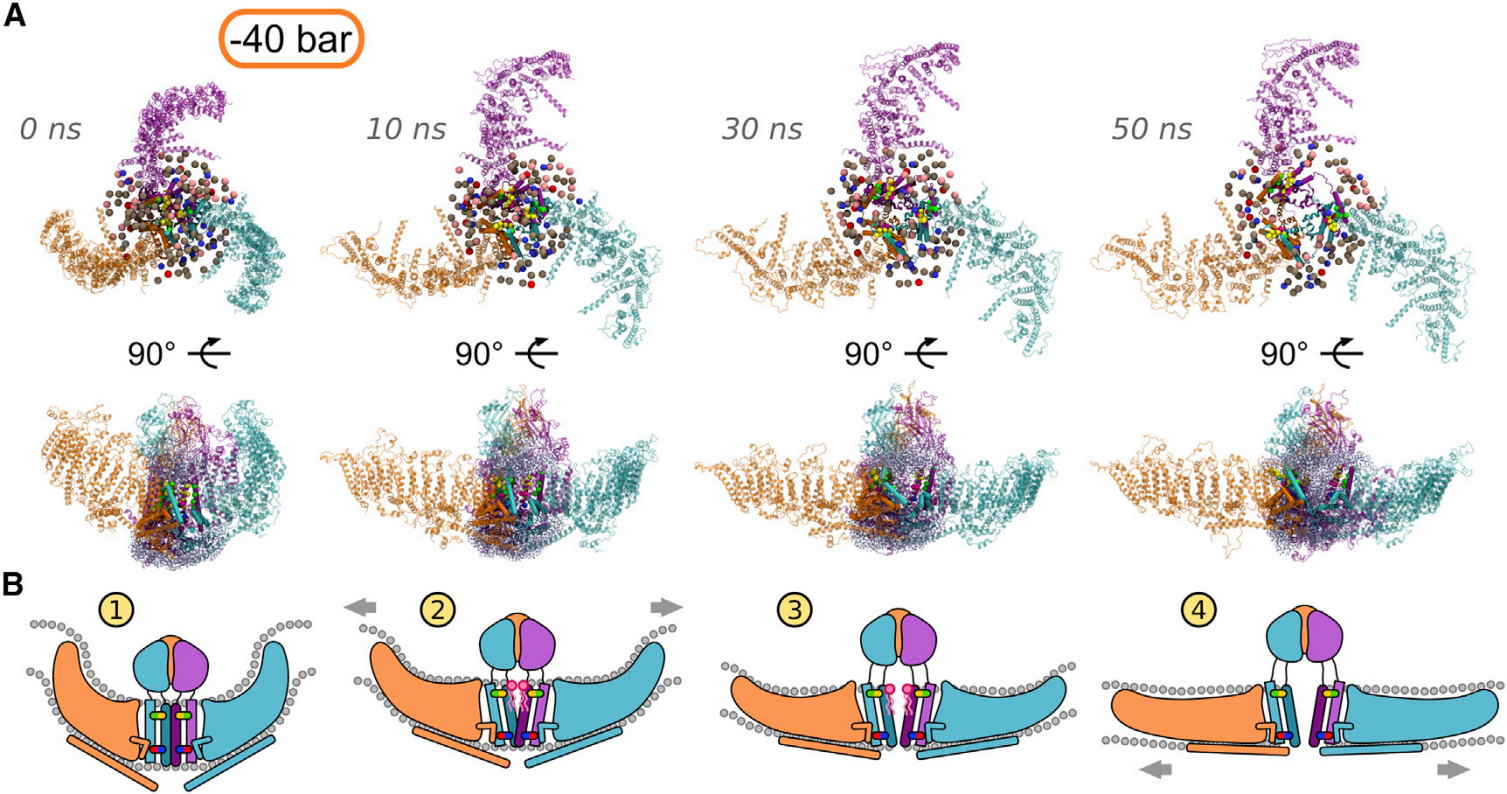
Proposed mechanism for the Piezo1 channel opening in response to membrane tension. (*A*) Snapshots from the second repeat simulation at −40 bar with the associated simulation time are shown. Membrane bilayer and solvent (i.e., water and ions) are not shown for clarity. The three Piezo1 subunits are indicated with different colors as in Fig. 1*A*. In the extracellular view at the top, the CED has been removed for clarity. Phosphorous and oxygen atoms from lipid headgroups and cholesterol, respectively, are shown (lipids are within 20 Å from the residues selected for the calculation of the cavity; see Materials and methods). The color code is as follows: POPC, tan; POPE, pink; POPS, red; PIP_2_, gray; and cholesterol, violet. The C*α* atoms from interacting residues between the helices TM37 and TM38 and the elbow are indicated as spheres as in Fig. 3*E*. In the Piezo1 structures in the middle panel, water molecules within 8 Å from the residues selected for the calculation of the cavity (see Materials and methods) are shown as light cyan transparent spheres. (*B*) Given is a cartoon representation explaining the Piezo1 activation mechanism coupled to membrane tension. The color code is the same as in (*A*). The hydrophobic interactions between the TM37 and TM38 and the salt bridge between TM38 and the elbow from a neighboring subunit are indicated in yellow/green and in blue/red, respectively. Lipids that initially occupy the Piezo1 channel mouth and progressively move away from it with the increase tension are schematically depicted in pink. Membrane tension and Piezo1 blade expansion are indicated by gray arrows. To see this figure in color, go online.

We also found that lipid and cholesterol molecules initially overlap with this volume, and therefore, they are initially within the Piezo1 pore region when the channel is closed, but they increasingly leave the cavity as higher tension is applied to the bilayer (Fig. 4 *A*). The three independent system set-ups followed by as many CG equilibrations demonstrate that lipids get within the channel mouth during the equilibration phase. This demonstrates that there is enough space in this closed structure of Piezo1 for lipids to occupy the channel mouth. This may also suggest that the presence of lipids in the pore may be required to maintain the channel mouth closed (Fig. S5). Indeed, a higher tension increases the APL in the proximity of the channel mouth and results in higher volume in the pore cavity because it enables lipids and cholesterol to move outside of the cavity in addition to a larger movement of the pore-lining helices TM37 and TM38. This hypothesis may explain biochemical data that suggest a functional role of lipids in Piezo1 regulation (24,48,49).

Analysis of the lipid distribution around the protein (Fig. S8) shows that POPE and POPC lipids in the simulations with stretch applied to the membrane tend to reside within the channel mouth in agreement with our previous work (50). Therefore, our simulations suggest that the tension applied to the bilayer during functional activation results not only in the displacement of TM38 and TM37 but also in the movement of lipids away from the Piezo1 pore. These two events occur simultaneously and may both be required for Piezo1 full opening.

An open question is how the mechanical stimuli are transferred from the blades to the channel pore. Our results suggest that the flattening and expansion of the blades is transmitted to the TM helices 37 and 38, guiding the pore opening. Noticeably, the pore region of the homologous Piezo2 has a similar structure (43), suggesting a similar mechanism. In Piezo1, the pore-lining TM38 is located antiparallel to the TM37. The latter, together with the elbow region (residues 2116–2142), a base helix (residues 2149–2175), and hairpin helices (residues 2501–2534) from all three subunits, create a cuff that encloses the pore-lining TM38 (Fig. 3 *E*; (11,14)). In all our simulations, we noticed two main pivotal interactions: the first is the interaction between the TM37 and TM38 via the hydrophobic residues Ile2466, Leu2469, Tyr2470, Ile2473, and Val2477, adjacent to the hydrophobic functional gate in TM38 involved in inactivation (Leu2475 and Val2476 (51)) and the residues Phe2198, Ile2202, Phe2205, and Phe2209 from TM37 (Fig. 3 *E*). Therefore, extension and expansion of the blades cause the displacement of TM37 that in turn “pulls” the pore-lining helix TM38 because of hydrophobic interactions. We suggest that this results in the opening of the channel and might explain why in the −40-bar simulation in which more expansion was observed, the pore was more open (Fig. 3 *E*).

The second pivotal interactions occur in a region toward the C-terminal end of TM37 and TM38. Previous work suggested the importance of the negative charge at position Glu2133 (Glu2416 in Piezo2) for conductance and ion selectivity (52). In particular, the single mutant E2133A showed half the unitary conductance with respect to the wild-type phenotype, and the negative charge from the glutamate has been suggested to allosterically modulate the selectivity of the Piezo1 filter (52). The Piezo1 cryo-EM structure maps the position Glu2133 in a helix named the elbow. Interestingly, the tip of each elbow points toward the TM38 of another Piezo1 subunit, and in our model, we found that Glu2133 on the elbows forms a salt bridge with the Arg2482 in a neighboring subunit in agreement with previous suggestions (Fig. 3 *E*; (12)). This salt bridge is retained in all our simulations between at least two Piezo1 chains with above 70% of occurrence, even after Piezo1 conformational rearrangement because of the applied negative pressure (Table S2). Therefore, we suggest that this interface is maintained during the conformational transition.

In agreement with this hypothesis, functional data (52) demonstrated that the mutant E2133D increases the conductance with respect to the wild-type, which is probably due to the short chain of the aspartate that would pull TM38 backward toward the elbow, thus reducing the pore constriction. Furthermore, this hypothesis rationalizes the effect of the less efficient mutant E2133K that was shown to decrease Piezo1 conductance (52). That is, the mutation to lysine opposite to the Arg2482 would push forward the TM38 because of repulsion, contributing to the increase of the channel constriction. In the E2133K mutant, the salt bridge is broken and so is the connection between the elbow and the TM38, which results in an impairment of the functional displacement connected to the blade expansion. In this condition, although less conducting, the channel is still functional (52), which may be due to the aforementioned hydrophobic interactions between TM37 and TM38 in synergy with the proposed lipid displacement. In conclusion, the predicted salt bridge between Glu2133 with Arg2482 in a neighboring subunit would provide an interaction interface between the Piezo1 chains so that each blade extension would control the gating of a nearby Piezo1 chain (Fig. 4 *B*). This may also provide a mechanism that ensures a prompt and mutual propagation of sensing between Piezo1 subunits.

## Conclusions

This study reveals possible molecular principles by which physiological Piezo1 channels may open in response to increased membrane tension. The suggested steps are shown in Fig. 4 *B* and are as follows: 1) without applied tension, Piezo1’s curved shape forces the bilayer to bend inwards, creating a stable indented trilobed topology with the CED hidden within it. In this resting conformation, the Piezo1 channel mouth is occupied by lipids. 2) Membrane tension on the bilayer results in the flattening and in-plane expansion of its blades and tilting of its beam helices. 3) Piezo1 expansion and flattening allows the blades to “pull” helices 37 and 38 via hydrophobic interactions to tilt and progressively move away from the channel pore, opening the Piezo1 channel. And 4) in the open conformation, Piezo1 blades are fully extended and flat, CED is exposed, and beam helices are almost parallel to the lipid bilayer. The elbow regions from neighboring subunits pull the pore-lining helices, TM38. The increase in the APL due to tension allows displacement of the lipids that occupy the channel mouth, resulting in an open conformation. Therefore, both conformational changes and increase in the APL that allow movement of lipids away from the channel pore region might be required for Piezo1 channel ion pore opening. Our studies also demonstrate that it is the blade region that senses the tension in the membrane and that membrane thinning is not sufficient to activate Piezo1 without the presence of tension.

Recent structural data showed that the second member of this family of proteins, Piezo2, has a similar shape with Piezo1. However, Piezo1 and Piezo2 have rather low sequence similarity, and Piezo2 is larger. Additionally, Piezo2 is likely to create a deeper dome in the membrane (∼9 nm compared with ∼6 nm of Piezo1) and have a larger footprint because of its larger size. The two channels also have differences in the topology of their pore region. Therefore, although it is possible that the mechanism presented here may be similar for Piezo2, further studies are needed to examine whether differences in Piezo1 and Piezo2 will result in differences in activation and sensitivity to tension.

A recent study has also suggested that other activation mechanisms may be possible for Piezo1 (53,54). In particular, Piezo1 was suggested to be an RNA sensor for single-strand RNA (ssRNA) (53). It is still unclear whether RNA binds to Piezo1, and thus, it is not possible to know how ssRNA might activate the channel. However, our recent work predicts strong interactions between Piezo1 and anionic phospholipids present exclusively in the inner leaflet of the membrane (50). It may be possible that the ssRNA backbone negative charge might have a potential similar role to anionic lipids on Piezo1 dynamics, but further studies are needed to understand how ssRNA activates Piezo1.

It has also been shown that the small molecule Yoda1 can activate Piezo1. A recent study proposed a molecular-wedge mechanism for Yoda1-mediated Piezo1 activation (15). Therefore, it is possible that functional Yoda1 binding sites might become available during the conformational changes associated to the flattening observed in our study. Moreover, Yoda1 is a hydrophobic molecule, and thus, it is likely to interact with the membrane bilayer, possibly altering the thickness and/or the membrane curvature. These alterations could have a local effect in the proximity of the protein affecting Piezo1 flattening. Further studies are needed to clarify the molecular mechanism of how Yoda1 may activate Piezo1.

Although this model informs ideas about the Piezo1 channel’s response to force and its consequent opening and it agrees with previous experimental evidence and hypotheses, it nevertheless has potential limitations that should be considered. The modeled channel is a fragment devoid of three N-terminal bundles in each Piezo1 and long, probably unstructured, cytoplasmic loop regions. Whereas the latter are still poorly annotated, Piezo1 blades have been associated with pressure sensing, thus at this stage, we are not able to fully extend the model to the complete protein.

Additionally, as mentioned above, usage of nonphysiological pressure or temperature may induce some caveats. Moreover, Piezo1 is activated by shear stress along the membrane surface, an important component not present in our model, which could be additive to the stretch in vivo. Further, Piezo1 functions at the millisecond timescale, which in conjunction with the recorded activating pressure, led us to a further limitation when using computer simulations. However, we were able to accelerate the process and investigate for the first time, to our knowledge, not only a flatten Piezo1 but intermediate conformations from the closed to open state within a simulated native lipid environment.

Despite the considerations above regarding pressure dynamics, we believe that this study informs how the fascinating fold and shape of Piezo1 channel might allow this protein to sense and transduce mechanical signals throughout the membrane, and we hope it will help to pave the way for future investigation once the Piezo1 full-length structure becomes available.

## Author contributions

The research was designed by D.D.V., D.J.B., and A.C.K. D.D.V. performed the research and acquired and analyzed all the data. A.C.K. and D.J.B. supervised the project. D.D.V., D.J.B., and A.C.K. wrote the article.

## Supporting material

Document S1. Figs. S1–S8 and Tables S1–S2

Document S2. Article plus supporting material
