# Supplementary material for: Molecular dynamics simulations of Piezo1 channel opening by increases in membrane tension
Source: Biophys J. 2021 Feb 12;120(8):1510–21. doi: 10.1016/j.bpj.2021.02.006 (PMC8105709; doi:10.1016/j.bpj.2021.02.006)
Supplement: Document S1. Figs. S1–S8 and Tables S1–S2 [file mmc1.pdf]

Biophysical Journal, Volume *120*

Supplemental information

Molecular dynamics simulations of Piezo1 channel opening by increases in membrane tension

Dario De Vecchis, David J. Beech, and Antreas C. Kalli

Molecular dynamics simulations of Piezo1 channel opening by increases in membrane tension

D De Vecchis, D J Beech and AC Kalli*

*Corresponding author

Supporting Material

Table S1. Composition of the simulated systems.

	Box size [nm] ($x \times y \times z$)	Protein residues	Lipid particles	Ions (Na, Cl, Ca)	Water particles	Total particles
Coarse-grained simulations						
Equil 1	31,33×31,33×27,97	4 680	37 413	4 467	183 811	236 593
Equil 2	31,40×31,40×27,85	4 680	37 485	4 465	183 780	236 632
Equil 3	31,40×31,40×27,86	4 680	37 485	4 467	183 809	236 663
Equil-Piezo1$^{\Delta1973}$	15,45×15,45×27,41	1 725	8 377	1 048	43 420	56 754
Equil-Piezo1$^{\Delta2104}$	13,96×13,96×29,31	1 332	7 355	916	38 076	49 356
Equil-Piezo1^{DLPC}	32,93×32,93×25,54	4 680	33 290	4 131	187 570	235 893
All-atom simulations						
Piezo1 1 bar	31,43×31,43×27,67	4 680	399 128	2 421, 2 192, 50	2 258 676	2 739 924
Piezo1 -5 bar	32,56×32,56×25,77	4 680	399 128	2 421, 2 192, 50	2 258 676	2 739 924
Piezo1 -20 bar	35,88×35,88×21,26	4 680	399 128	2 421, 2 192, 50	2 258 676	2 739 924
Piezo1 -30 bar	38,55×38,55×18,43	4 680	399 128	2 421, 2 192, 50	2 258 676	2 739 924
Piezo1 -40 bar (repeat 1)	41,30×41,30×16,07	4 680	399 128	2 421, 2 192, 50	2 258 676	2 739 924
Piezo1 -40 bar (repeat 2)	41,72×41,72×15,74	4 680	399 128	2 421, 2 192, 50	2 258 676	2 739 924
Piezo1$^{\Delta1973}$	19,67×19,67×16,87	1 725	89 385	584, 506, 12	533 454	651 952
Piezo1$^{\Delta2104}$	18,39×18,39×16,81	1 332	78 491	509, 442, 10	467 769	568 863
Piezo1^{DLPC}	33,31×33,31×24,62	4 680	352 874	1 931, 2 100, 50	2 300 262	2 734 674
Piezo1$^{-40.rep1}$	37,27×37,27×21,15	4 680	575 579	2 658, 2 254, 56	2 303 934	2 961 944
Piezo1$^{-40.rep2}$	37,18×37,18×21,30	4 680	573 093	2 663, 2 259, 56	2 309 355	2 964 889

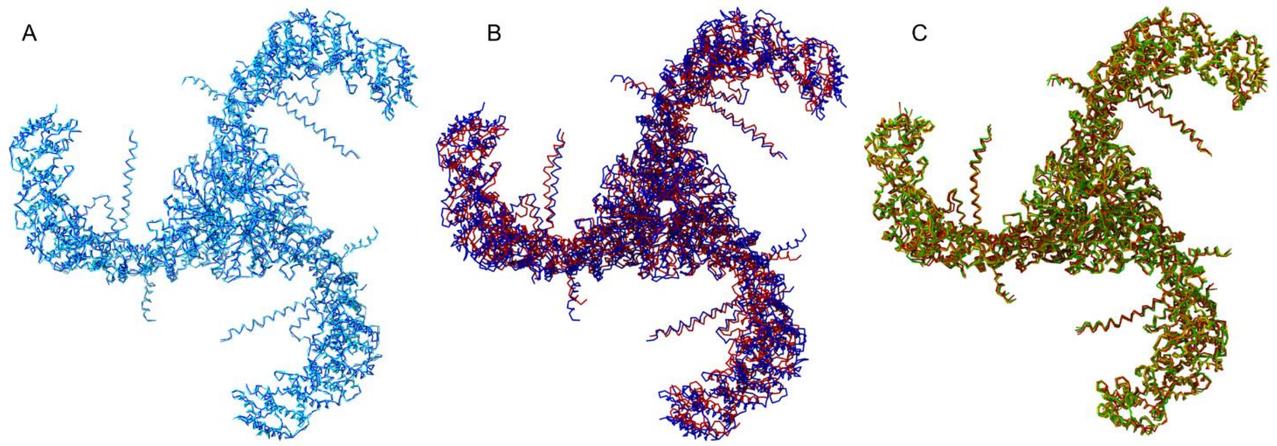

Figure S1. Structural comparison of the Piezo1 model during the equilibration phase. (A) The Cα trace of the Piezo1 structure after the initial modelling procedure (cyan) in comparison with the structure after the conversion in the coarse-grained representation (backbone beads are in blue). (B) Structural superposition between the Piezo1 structure after the conversion in the coarse-grained representation (backbone beads are in blue) and the structure after the minimisation in vacuum prior to the coarse-grained equilibration phase (backbone beads are in red). The RMSD is 4.07 Å. (C) Superposition between the structure after the minimisation in vacuum prior to the coarse-grained equilibration phase (backbone beads are in red) and the final snapshot from each of the independent three equilibration phases (backbone beads are in orange, yellow and green, respectively). For each of the three final snapshots, the RMSD is 1.19 Å, 1.14 Å and 1.20 Å, respectively.

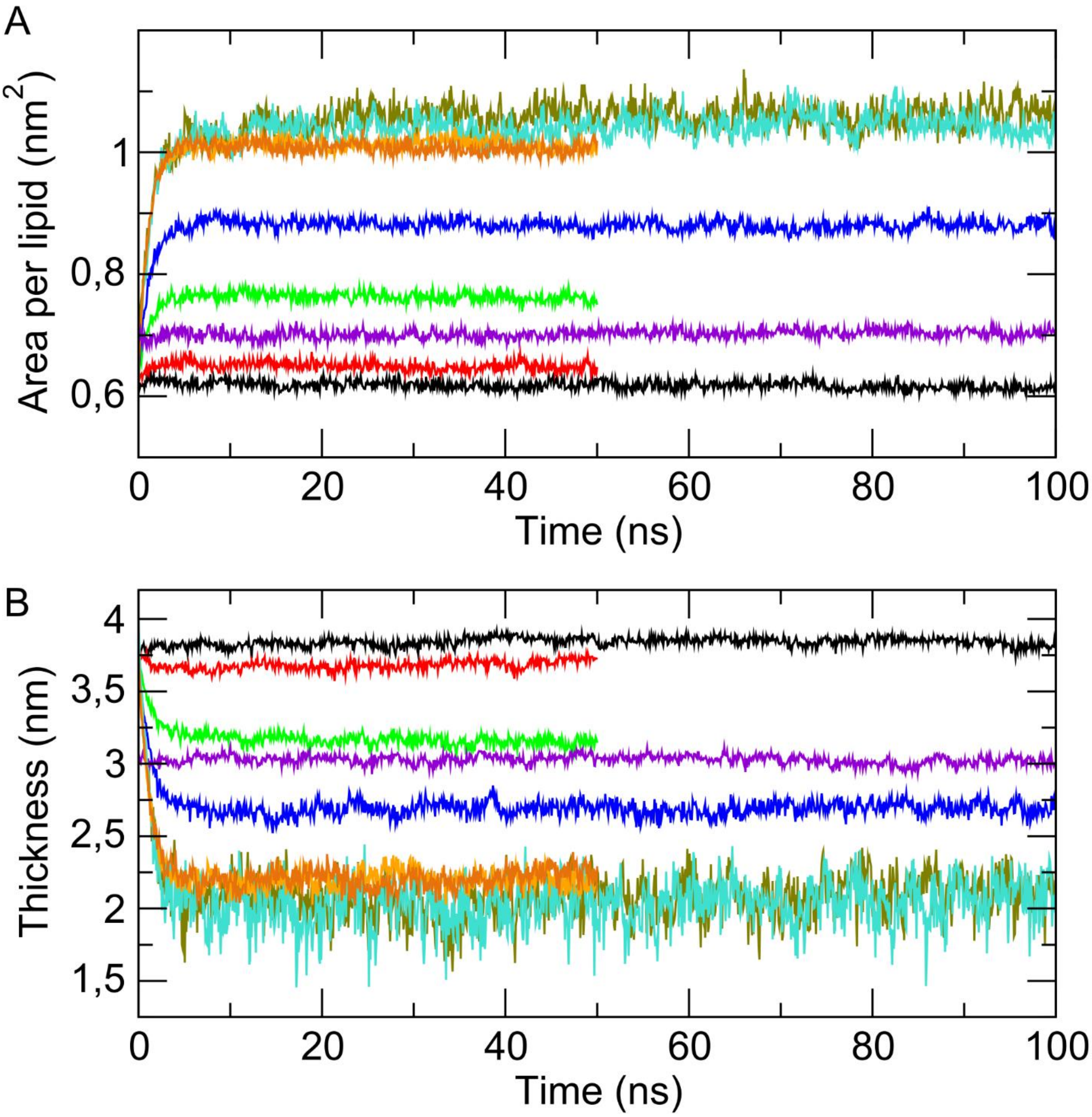

Figure S2. Area per lipid and membrane thickness during the simulations. For each system, the area per lipid (A) and the membrane bilayer thickness (B) in each simulation are indicated with different colours: black, 1 bar; red, -5 bar; green, -20 bar; blue, -30 bar; orange, -40 bar, Piezo1$^{\Delta 1973}$, tan; Piezo1$^{\Delta 2104}$, cyan; Piezo1^{DLPC}, purple.

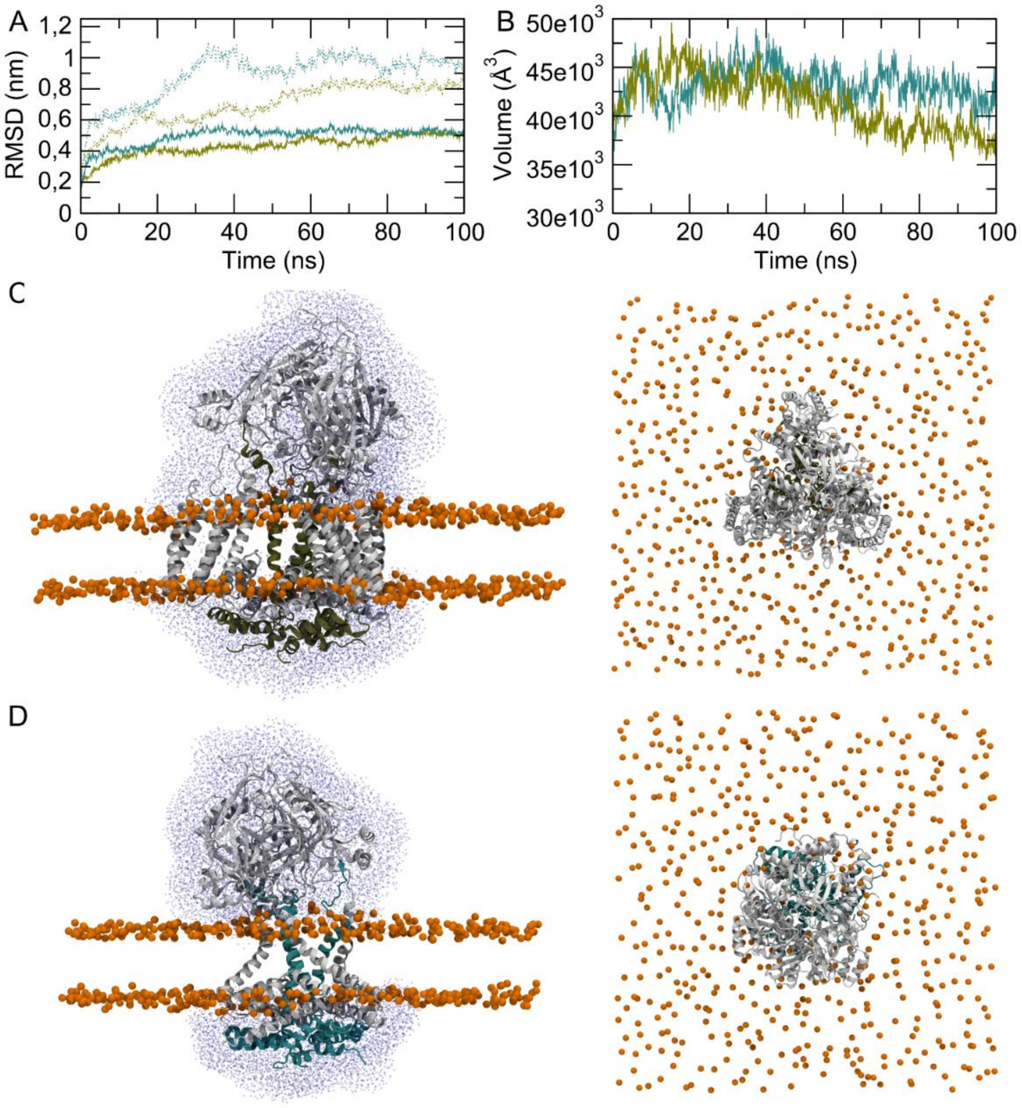

Figure S3. Piezo1 blade domain is required for mechanosensation. (A) Root mean-square deviation (RMSD) for the blade-free systems Piezo1$^{\Delta 1973}$ (tan) and Piezo1$^{\Delta 2104}$ (cyan) calculated using the Cα atoms of the protein (dashed lines) and the Piezo1 inner pore (solid lines). (B) Volume of the cavity of the Piezo1 pore region as a function of time for the blade-free simulated systems, the colour legend is the same as in A. The last frame from the 100 ns simulation at -40 bar pressure of Piezo1$^{\Delta 1973}$ (C) and Piezo1$^{\Delta 2104}$ (D). The Piezo1 inner pore is coloured as in A and B. Phosphate atoms from the phospholipids are represented as orange spheres. To indicate that the bilayer is devoid of water and the channel closed, only the water molecules (blue spheres) within 10 Å of the protein atoms are shown for clarity. For C and D, the top view is on the right.

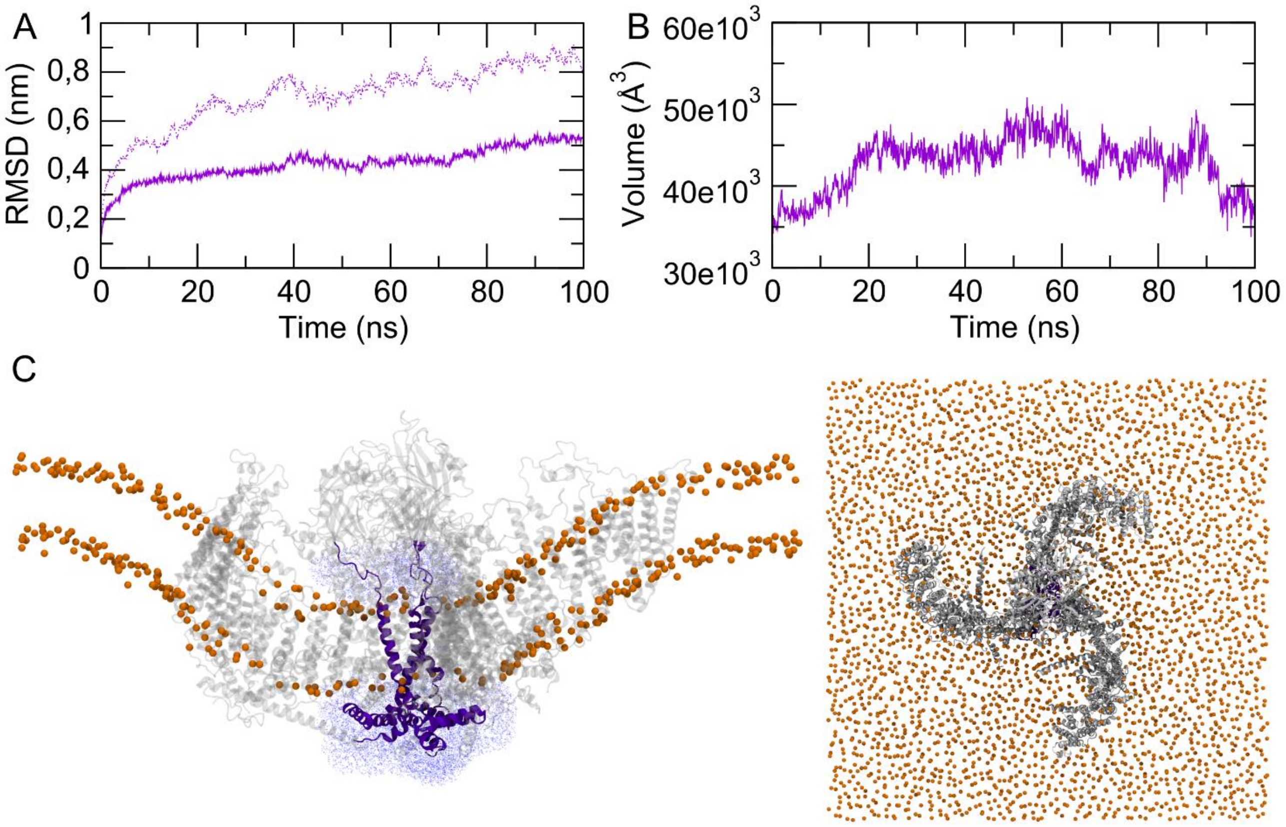

Figure S4. Simulations of Piezo1 embedded in a thinner bilayer that consists of 1,2-dilauroyl-sn-glycero-3-phosphocholine (DLPC) phospholipids. (A) Root mean-square deviation (RMSD) for the Piezo1^{DLPC} system calculated using the Cα atoms of the protein (dashed line) and the Piezo1 inner pore (solid line). (B) Volume of the cavity of the Piezo1 pore region as a function of time for the Piezo1^{DLPC} system. (C) The last frame from the 100 ns simulation at 1 bar pressure of Piezo1^{DLPC}. Extracellular view is on the right. The Piezo1 inner pore is coloured as in A (purple). The rest of the protein is displayed as transparent ribbon and phosphate atoms from the phospholipids are represented as orange spheres. Only a section of the membrane bilayer is shown here to appreciate the membrane indentation. To indicate that the bilayer is devoid of water and the channel closed, only the water molecules (blue spheres) within 10 Å of the Piezo1 pore region (purple) are shown for clarity.

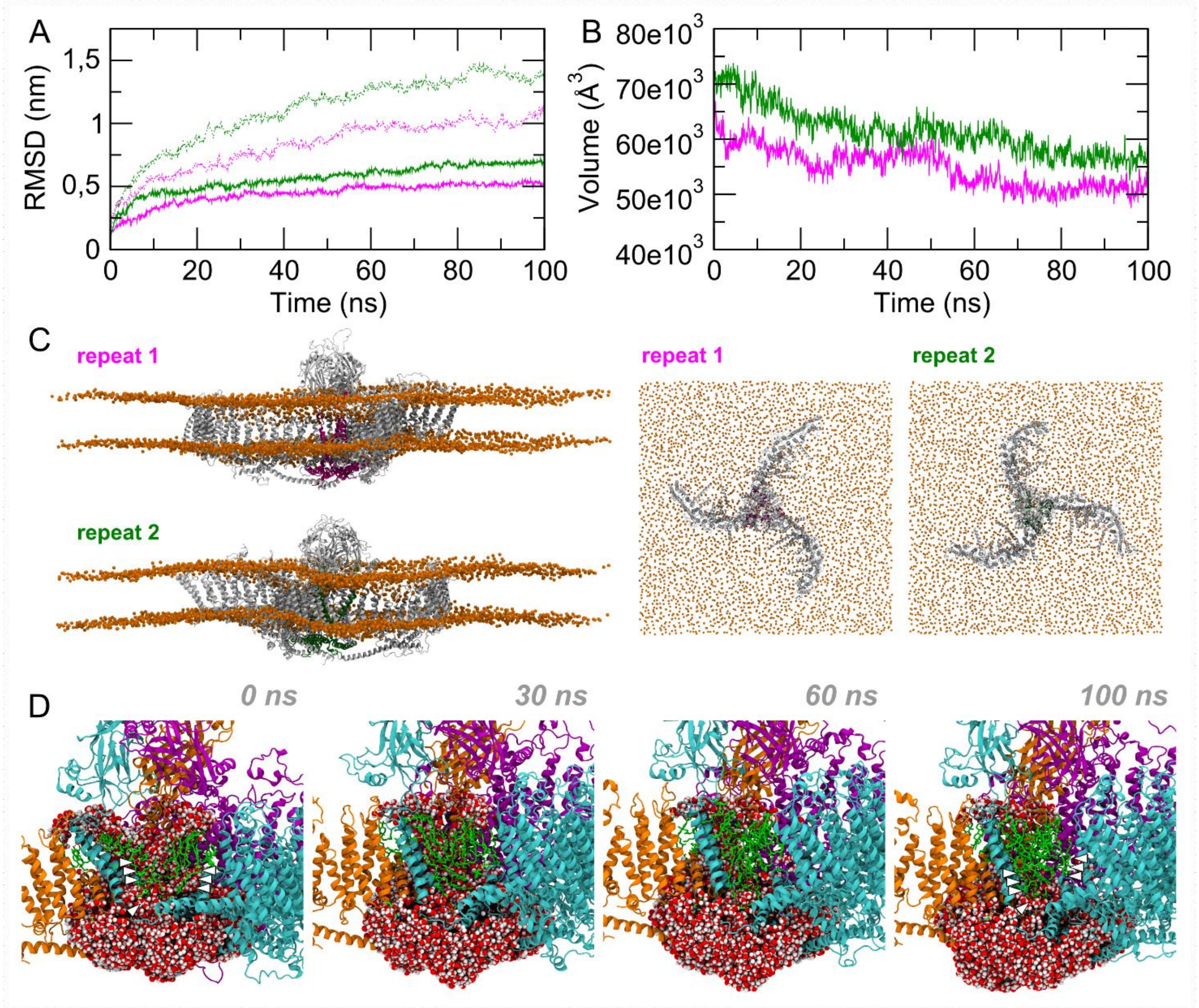

Figure S5 Tension ceasing causes lipids to occupy the Piezo1 channel mouth. (A) Root mean-square deviation (RMSD) for the systems Piezo1$^{-40.rep1}$ (magenta) and Piezo1$^{-40.rep2}$ (green) calculated using the Cα of the protein (dashed lines) and the Piezo1 inner pore (solid lines). (B) Volume of the cavity of the Piezo1 pore region as a function of time. The colour legend is the same as in A. (C) The last frame from 100 ns simulation at 1 bar pressure of Piezo1$^{-40.rep1}$ (repeat 1) and Piezo1$^{-40.rep1}$ (repeat 2). The Piezo1 inner pore is coloured as in A and B. Phosphorous atoms from the phospholipids are represented as orange spheres. The correspondent top views are shown on the right. (D) Sequential snapshots from the Piezo1$^{-40.rep2}$ simulations at 1 bar showing the progressive dehydration (arrows) of the Piezo1 pore caused by the absence of membrane tension and the phospholipids (green sticks) which progressively occupy the Piezo1 channel mouth.

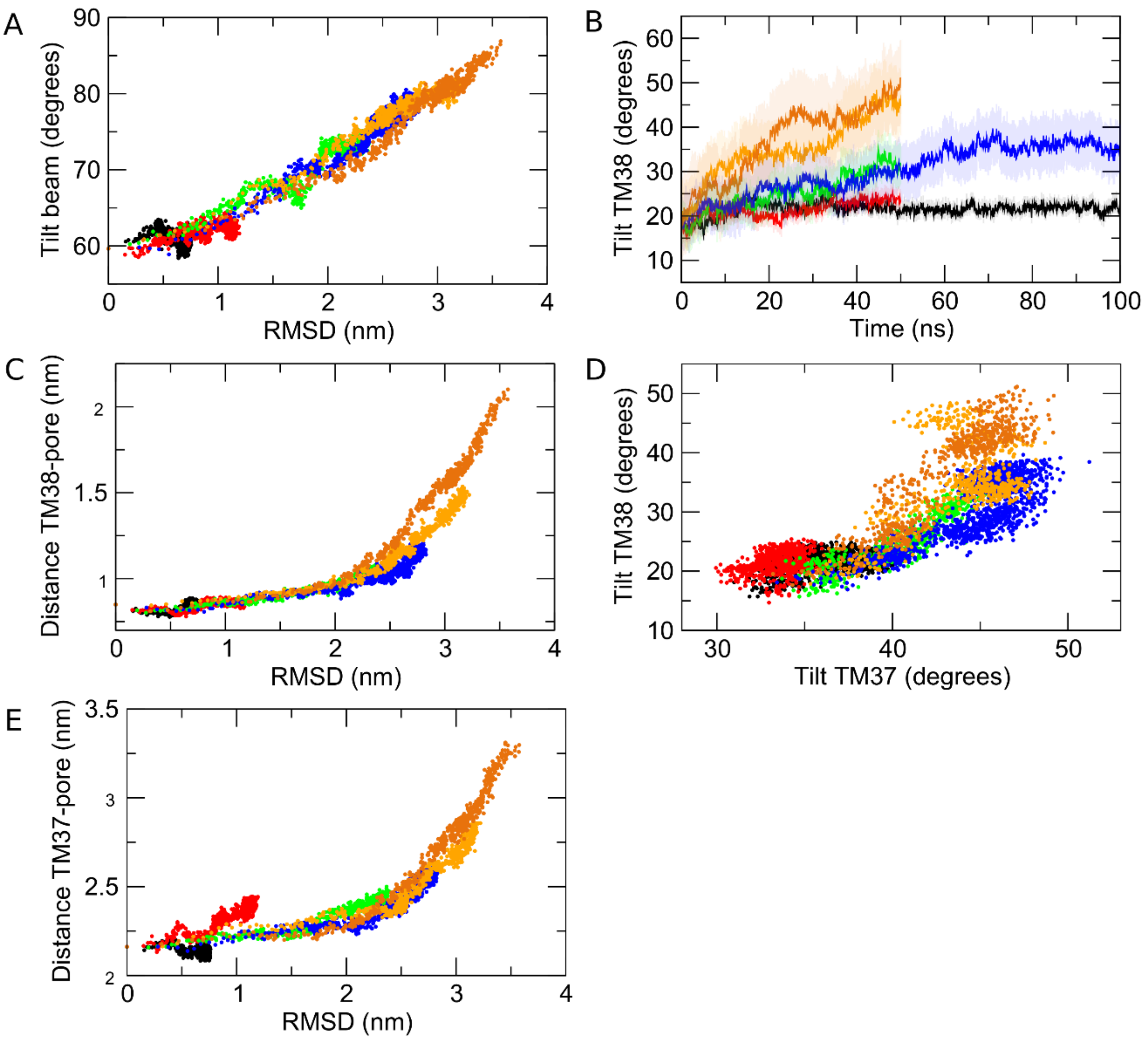

Figure S6. Correlation of Piezo1 pore-lining helices with changes in Piezo1 conformation. For each system, the applied negative pressure in each simulation is indicated with different colours: black, 1 bar; red, -5 bar; green, -20 bar; blue, -30 bar; orange, -40 bar. (A) Correlation between the root mean-square deviation (RMSD) of the Piezo1 blades and the tilt angle relative to the bilayer normal of the beam helix. (B) Tilt angle relative to the bilayer normal for the TM38 helix. (C) Correlation between the RMSD of the Piezo1 blades and the distance from the centre of mass of the TM38 helix and the Piezo1 pore. (D) Correlation between the tilt angle relative to the bilayer normal of the TM38 and TM37 helices. (E) Correlation between the RMSD of the Piezo1 blades and the distance from the respective centre of mass of the TM37 helix and the Piezo1 pore.

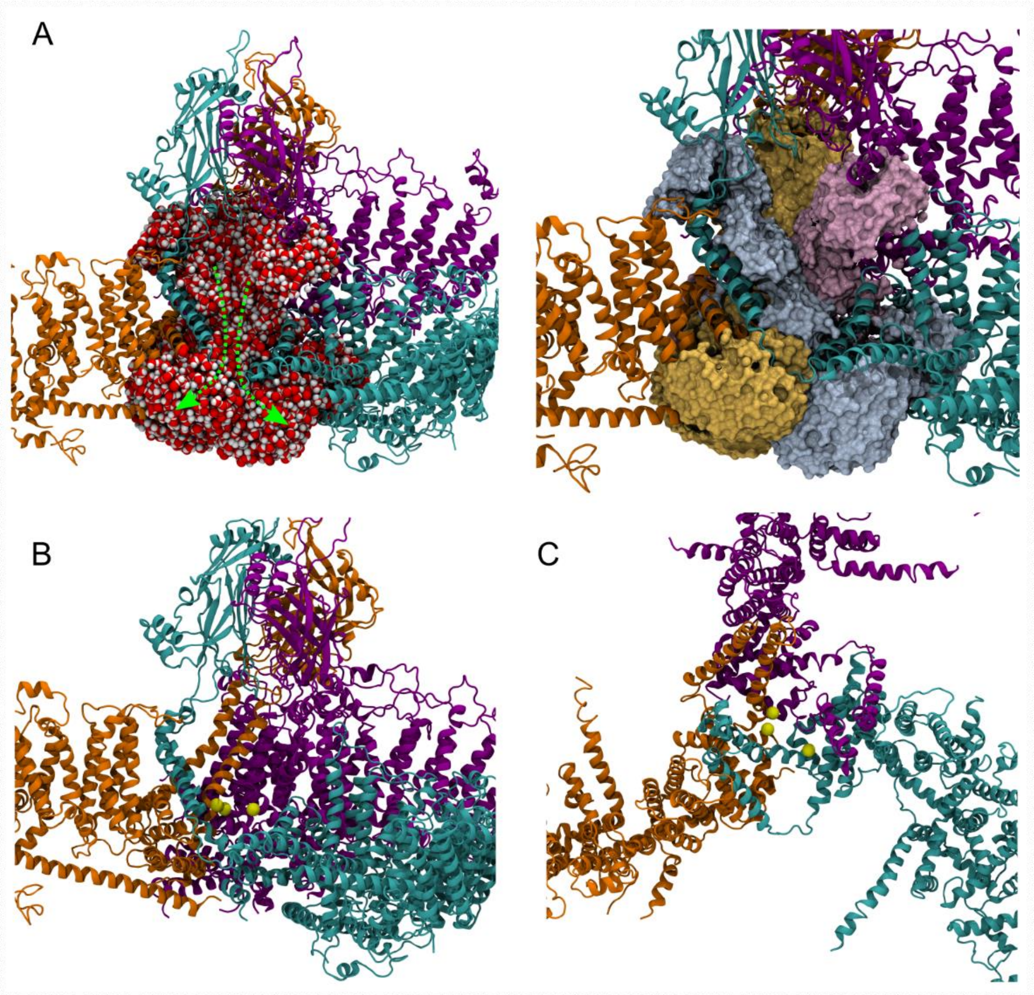

Figure S7. The open Piezo1 pore shows solvated lateral fenestrations and a pore cavity permeable to ions. (A) On the right, ribbon representation of Piezo1 channel from the last frame of the -40 bar system. Protein chains are coloured orange, cyan and purple. Water molecules within 10 Å of protein residues 2460-2547, that occupy the lateral fenestrations of the Piezo1 open pore are shown as van der Waals spheres. Arrows represent potential ion permeation pathways. On the left, the same frame as on the right is shown but the water molecules within 10 Å of residues 2460-2547 are shown as surfaces and depicted using a lighter colour which is similar with the correspondent protein chain. (B) Ribbon representation of Piezo1 channel from the last frame of the -40 bar system. Protein chains are coloured as in A. Three Sodium ions that occupy the Piezo1 open pore cavity are shown as yellow spheres. (C) Top view of B. The CED domain is omitted for clarity.

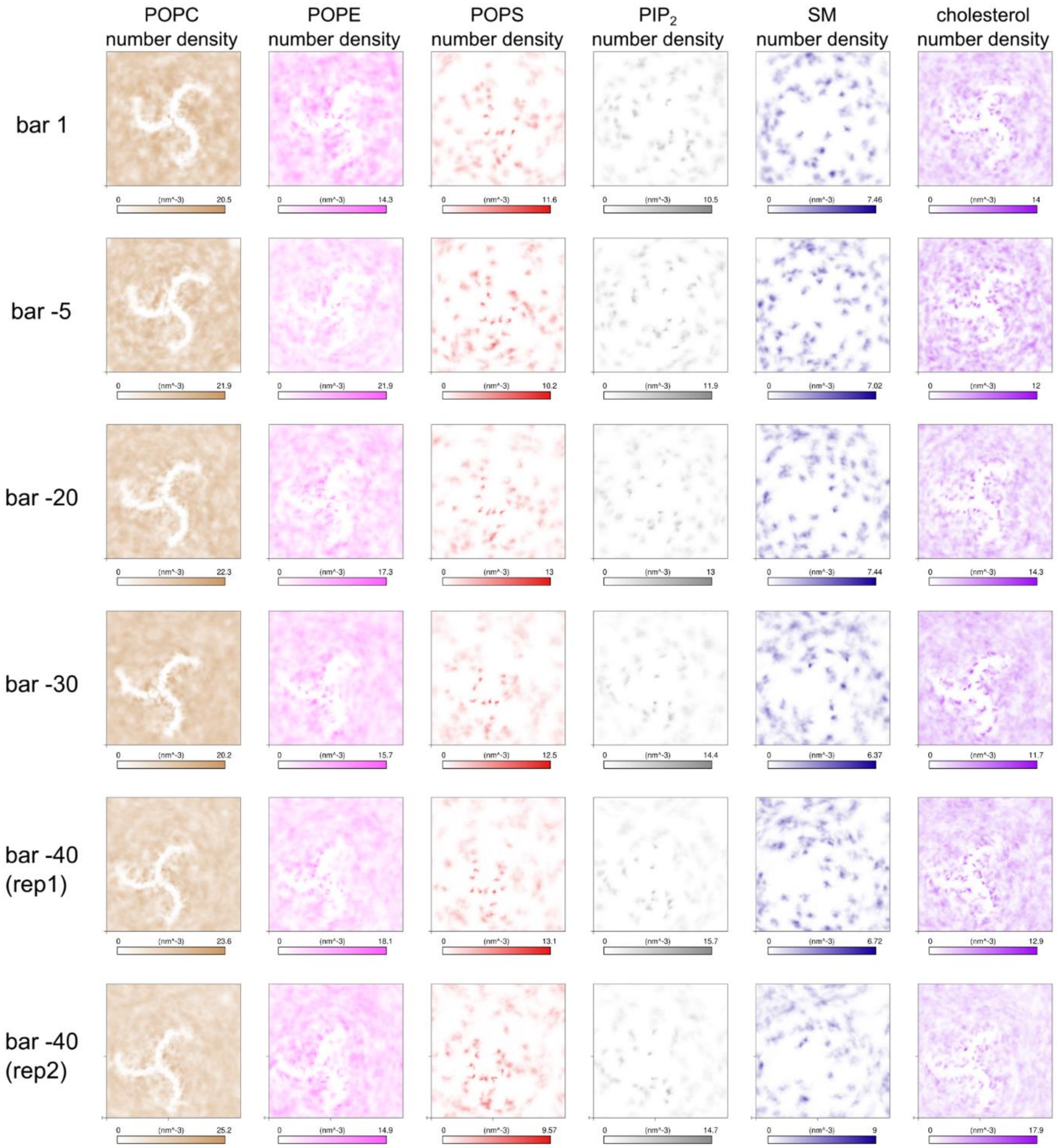

Figure S8. Lipid density maps. For each of the Piezo1 simulated system indicated on the left, the 2D lipid density maps are shown. Colours are: 1-palmitoyl-2-oleyl-phosphtidylcholine (POPC), tan; 1-palmitoyl-2-oleyl-phosphtidylethanolamine (POPE), pink; 1-palmitoyl-2-oleyl-phosphtidylserine (POPS), red; phosphatidylinositol 4,5-bisphosphate (PIP_2), grey; sphingomyelin (SM), blue; cholesterol, violet.

Table S2. Predicted salt bridge between the elbow and the pore-lining helix 38

	+1 bar	-5 bar	-20 bar	-30 bar	-40 bar (rep1)	-40 bar (rep 2)
E2133.a - R2482.c	100	100	100	100	100	100
E2133.a - R2482.b	98	98	100	96	70 (98)	100 (100)
E2133.b - R2482.c	100	100	100	7 (62)	8 (89)	7 (83)

When occurrence was lower than 70%, numbers between parentheses indicate occurrence within the first 30 ns of simulations.

Movie S1. Stretch molecular dynamics simulation causes flattening of the Piezo1 membrane indentation. The first repeat of the -40 bar simulation is shown here. The Piezo1 chains are indicated in ribbon representation and coloured in orange, purple and cyan. Phosphate atoms from the phospholipids are depicted as blue spheres. Solvent molecules have been removed for clarity.

Movie S2. Stretch molecular dynamics simulation causes the in-plane area expansion of Piezo1. The top view of the first repeat of the -40 bar simulation is shown here. The Piezo1 chains are indicated in ribbon representation and coloured in orange, purple and cyan. Phosphate atoms from the phospholipids are depicted as blue spheres. Solvent molecules have been removed for clarity.
